# Can long-term care insurance pilots promote urban industrial structure upgrading? Evidence from a quasi-natural experiment using the China Family Panel Studies

**DOI:** 10.3389/fpubh.2026.1962465

**Published:** 2026-08-28

**Authors:** Jiyang Li, Liangyu Chen

**Affiliations:** 1 School of Economics and Management, Wuhan University, Wuhan, China; 2 School of Tourism and Hospitality Management, Wuhan City Polytechnic, Wuhan, Hubei, China

**Keywords:** household financial asset allocation, industrial structure upgrading, labor supply, long-term care insurance, multiperiod difference-in-differences

## Abstract

Against the backdrop of population aging and rising demand for disability-related care, long-term care insurance (LTCI) not only shares household care risks but may also reshape urban economic structure by expanding formal care services. This study examines whether China’s LTCI pilots promote urban industrial structure upgrading and explores the associated transmission channels. Treating the staggered rollout of China’s LTCI pilots as a quasi-natural experiment, we match the 2014-2020 China Family Panel Studies with city-level macroeconomic data and estimate a multiperiod difference-in-differences model with individual- and city-level controls, individual and year fixed effects, and city-clustered standard errors. We further apply the Callaway-Sant’Anna estimator to account for staggered adoption and heterogeneous treatment effects. The preferred specification yields an LTCI coefficient of 0.1860, equivalent to approximately 11.3% of the sample mean of the industrial structure upgrading index. The Callaway-Sant’Anna overall average treatment effect on the treated is 0.1974, confirming the baseline finding. The effect is stronger in cities with greater government intervention, in eastern China, and in cities with more developed financial systems. Mechanism analysis indicates that LTCI increases household members’ employment probability and improves household financial product allocation. These findings suggest that LTCI can promote health and eldercare services and related modern service industries through formal care expansion, released household labor supply, and improved household capital allocation, indicating that the effects of LTCI extend beyond household welfare and healthcare to urban industrial transformation.

## 1 Introduction

Population aging is reshaping household risk exposure and demand for public services. Unlike ordinary health shocks, disability is typically persistent and care intensive, and it generates substantial costs outside the formal health system. Existing evidence shows that sustained caregiving responsibilities reduce labor force participation and increase withdrawal from paid employment (1), while informal care affects households through healthcare use, opportunity costs, and labor-market participation (2–4). Evidence from China further indicates that health risks increase household financial vulnerability (5). As family size declines, population mobility accelerates, and female labor force participation rises, a stable and sustainable system for financing and delivering long-term care has become increasingly important.

LTCI socializes part of the cost of basic daily assistance and related nursing care for people with disabilities. Its objectives extend beyond relieving the household care burden to standardizing disability assessment, fostering professional care providers, and establishing a multilevel service network. Several cities conducted local experiments before the national pilot program. In 2016, the Ministry of Human Resources and Social Security launched the first national pilot program, and in 2020 the National Healthcare Security Administration and the Ministry of Finance expanded the program, moving the reform from local experimentation toward institutional development (6, 7).

The existing literature evaluates LTCI mainly in terms of healthcare utilization, informal care burdens, older adults’ health, labor supply, and household welfare. Studies find that LTCI reduces hospital use and medical expenditures (8, 9), lowers unmet care needs, informal care intensity, and out-of-pocket spending while improving older adults’ health (10), and weakens precautionary saving induced by long-term care risk while increasing nonmedical consumption (11). Evidence from Japan and China also shows that formal care and LTCI raise caregivers’ employment probability and labor supply (12–14). These studies provide a strong household-welfare perspective, but the implications of LTCI for urban industrial structure remain less developed.

LTCI also connects social protection, public health service provision, and service-sector development. Insurance payments convert care needs that were previously absorbed within households into identifiable and effective market demand, thereby expanding nursing care, rehabilitation, community care, and health management services. Evidence based on Chinese city-level registration and firm-entry data shows that LTCI increases the number and registered capital of care providers and stimulates entry by long-term care service firms (15, 16). This process improves access to continuous care for people with disabilities while reallocating capital, labor, and demand toward health, eldercare, and related modern services. The resulting structural change is consistent with theories of structural transformation and the rise of the service economy (17, 18) and constitutes an important public health spillover of LTCI.

The conversion of insurance payments into new service capacity depends on local institutions and market conditions. LTCI requires coordination among healthcare security, civil affairs, public health, and fiscal authorities. Research on state capacity and local governance shows that implementation capacity affects how effectively public policy supports markets, allocates resources, and responds to local needs (19, 20). Eastern cities generally possess stronger care infrastructure, a larger pool of professionals, and more mature service markets. Financial development also determines whether care providers and health-service firms can obtain long-term funding; prior studies show that it alleviates external financing constraints, improves capital allocation, and supports innovation (21–23). Government capacity, regional development, and financial development may thus condition the structural effects of LTCI.

This study treats the LTCI pilot program as a quasi-natural experiment and matches individual and household information from the China Family Panel Studies (CFPS) with city-level indicators and policy timing. Because conventional two-way fixed-effects estimates may be affected when treatment effects differ across staggered policy cohorts (24, 25), the analysis also applies the Callaway–Sant’Anna group-time average treatment effect estimator and compares its results with the baseline estimate. The baseline specification includes individual and year fixed effects and clusters standard errors at the city level, where policy assignment occurs (26).

The study makes three contributions. First, it extends research on the economic consequences of LTCI from healthcare use and household welfare to urban industrial transformation, thereby identifying a public health spillover operating through health and eldercare service supply. Second, it combines individual, household, and city information and uses both a conventional multiperiod difference-in-differences model and the Callaway–Sant’Anna estimator to reduce concerns arising from staggered adoption and heterogeneous treatment effects. Third, it identifies released labor supply and improved household financial asset allocation as two transmission channels and examines government intervention, regional location, and financial development as conditions shaping the conversion of insurance payments into additional service supply.

## 2 Institutional background and theoretical analysis

### 2.1 Institutional background

China’s LTCI reform has evolved gradually from local experimentation to national pilots. Several localities first explored disability assessment, benefit payments, financing arrangements, and provider management. The 2016 national pilot established a basic institutional framework, and the 2020 expansion broadened the reform’s geographic coverage. Although financing sources, eligible populations, payment methods, and covered services continue to vary across cities, the programs share a central objective: purchasing formal care services, regulating service provision, and reducing household care burdens (27, 28). The resulting differences in adoption timing provide the temporal and geographic variation used in the empirical analysis.

The policy date is defined as the year in which a city formally established or implemented LTCI. When implementation occurred between two CFPS waves, the next observable survey wave is treated as the first post-policy observation. Pilot cities and implementation dates are identified from the 2016 national guidance, the 2020 expansion guidance, and implementation documents issued by local healthcare security authorities, and are matched to prefecture-level city codes.

### 2.2 Long-term care insurance promotes industrial structure upgrading

LTCI promotes industrial structure upgrading by marketizing care demand. Without socialized payment, disability care is often provided as unpaid household labor or informal services and does not generate stable market demand. Insurance increases households’ ability to purchase formal services and shifts care from the household sector to nursing institutions, community providers, and medical rehabilitation organizations. The observed entry of nonresidential care firms following LTCI adoption demonstrates that institutional payments can transform household needs into an expandable service market and raise the output and employment share of the tertiary sector (15).

LTCI also supports structural upgrading through specialization and interindustry linkages. Designated-provider management, service catalogues, and quality regulation promote standardization and professionalization. Additional demand stimulates related activities such as rehabilitation equipment, age-friendly renovation, care training, health information services, and commercial insurance. Input–output evidence indicates that the health industry has strong linkages with medium-technology manufacturing and producer services and that final demand is an important driver of its development (29). Growth in care demand can therefore generate broad structural effects along the health-industry value chain.

In addition, LTCI reduces uncertainty surrounding disability risk and precautionary saving, relaxes household caregiving time constraints, and improves the allocation of labor and household capital. Expected medical expenditures are an important source of precautionary saving among older households (30, 31), whereas LTCI increases nonmedical consumption (11). Formal care also releases caregivers’ labor time (32, 33). Together, these channels direct productive factors and household resources toward market-based service activities.

*H1: LTCI pilots significantly promote urban industrial structure upgrading.*

### 2.3 Long-term care insurance releases household labor supply

Disability care is time intensive, and family caregiving responsibilities reduce the time available for market work. By purchasing institutional, community-based, and home-based formal care, LTCI transfers part of care provision from households to professional service providers and relaxes caregivers’ time constraints. Evidence from Japan and China shows that formal care and LTCI increase caregivers’ employment and labor supply (12–14, 32, 33). Released labor both raises household members’ probability of entering market employment and supplies workers to expanding nursing, rehabilitation, and health-management industries, thereby reallocating labor from the household sector to the market and from traditional activities to modern services.

*H2*: *LTCI pilots promote urban industrial structure upgrading by releasing household labor supply.*

### 2.4 Long-term care insurance improves household financial asset allocation

Long-term care risk increases uncertainty over future household expenditure and strengthens precautionary saving in liquid assets. LTCI converts part of unpredictable care expenditure into institutional protection and stabilizes expectations regarding future medical and care costs, creating scope for households to adjust their financial portfolios (11, 30, 31). A more diversified portfolio improves intertemporal capital allocation and raises demand for wealth management, insurance, payment, and other modern financial services. At the same time, funds mobilized through financial intermediation can support investment by nursing, rehabilitation, and health-management providers. Improved household financial asset allocation can therefore connect risk sharing with the financing and expansion of health and other modern service industries.

*H3: LTCI pilots promote urban industrial structure upgrading by improving household financial asset allocation.*

### 2.5 Urban supply conditions strengthen the structural effect of long-term care insurance

The structural effect of LTCI also depends on a city’s capacity to convert institutional payments into new service supply. Government coordination improves financing, assessment, provider entry, and supervision (19, 20). Stronger regional economic and public-service foundations facilitate the realization of care demand and service-sector upgrading (34, 35). Greater financial development alleviates financing constraints faced by care providers and health-service firms and improves capital allocation (21–23). Together, these conditions accelerate the conversion of insurance payments into provider entry, service expansion, and industrial investment.

*H4: The positive effect of LTCI pilots on industrial structure upgrading is stronger in cities with greater government coordination capacity, in eastern China, and in cities with higher financial development.*

## 3 Materials and methods

### 3.1 Model specification

The staggered implementation of LTCI across cities is used to estimate the following multiperiod difference-in-differences model:

ISUct=α+βLTCIct+γXict+μi+λt+εict

where ISUct is the industrial structure upgrading index for city c in year t, measured as the ratio of tertiary- to secondary-industry value added; LTCIct equals one for a pilot city in the first observable policy-affected survey wave and thereafter and zero otherwise; Xict contains individual-, household-, and city-level controls; and μi and λt denote individual and year fixed effects, respectively. Individual fixed effects absorb time-invariant ability, household endowments, and long-run residential characteristics, while year fixed effects control for shocks common to all cities. The coefficient *β* is the average absolute change in the sectoral ratio after policy implementation. Because treatment and the outcome vary at the city level, standard errors are clustered by city to allow arbitrary within-city correlation.

### 3.2 Data sources and sample construction

Individual and household data are drawn from the 2014, 2016, 2018, and 2020 waves of the CFPS. City-level industrial structure and macroeconomic indicators are compiled from the China City Statistical Yearbook, provincial and municipal statistical yearbooks, and statistical communiqués on national economic and social development. The city database contains GDP per capita, urbanization, GDP growth, financial development, and informatization indicators.

LTCI policy information, city-level data, and CFPS records are matched using city codes. Observations with missing core variables are removed, and individuals observed only once are excluded from the fixed-effects sample. The complete-control sample contains 1,231 person-year observations, 494 individuals, and 73 cities. Because complete city-level controls for 2022 were unavailable, the baseline period ends in 2020. Thirteen LTCI pilot cities are represented in the baseline sample.

### 3.3 Variables

The dependent variable is the industrial structure upgrading index (industry_upgrade), measured as the ratio of tertiary-industry value added to secondary-industry value added. A one-unit increase means that the tertiary-to-secondary value-added ratio rises by one unit; it indicates a stronger relative position of services but does not mean that tertiary-industry value added itself grows by the same percentage. This measure captures the intersectoral reallocation of output emphasized in structural-transformation research (36).

The core explanatory variable is the LTCI pilot indicator (ltci_did), which equals one when a city has entered the pilot program and the survey year is no earlier than the implementation year, and zero otherwise. It therefore combines cross-city treatment status with policy timing.

Individual and household controls include squared age of the household head, years of education, marital status, agricultural household registration, household size, number of older household members, employment status, and the logarithm of total household income. City-level controls include the logarithm of GDP per capita, urbanization rate, GDP growth rate, financial development, and informatization. The industrial structure index itself is excluded from the control set to avoid mechanical correlation and over-control.

Government intervention and financial development are measured using their pre-policy city values in 2014 to reduce reverse causality and post-treatment bias. Regional location is classified according to whether the city is in eastern China. Continuous interaction variables are standardized. Descriptive statistics are reported in Table 1.

**Table 1:** Descriptive statistics.

Variable	*N*	Mean	SD	Min	Max
Industrial structure upgrading index	1,231	1.6422	0.7015	0.6837	5.2968
LTCI pilot	1,231	0.3298	0.4703	0	1
Age squared/100	1,231	26.9165	12.9402	4	81
Years of education	1,231	12.6296	3.3967	0	19
Married	1,231	0.8700	0.3364	0	1
Agricultural hukou	1,231	0.1909	0.3932	0	1
Household size	1,231	3.1673	1.2737	1	11
Older household members	1,231	0.4200	0.7156	0	3
Currently employed	1,231	0.6621	0.4732	0	1
Log household income	1,231	11.6434	0.9835	0	14.5087
Log city GDP per capita	1,231	11.3462	0.5177	9.6711	12.0684
Urbanization rate	1,231	0.7879	0.1343	0.4312	0.9283
City GDP growth rate	1,231	0.0596	0.0354	−0.2063	0.1112
City financial development	1,231	4.1288	1.6316	1.1486	7.6203
City informatization	1,231	0.0258	0.0144	0.0054	0.1570

Descriptive statistics are calculated using the common sample that contains the full set of controls and enters the individual fixed-effects model.

## 4 Results

### 4.1 Baseline results

Table 2 reports the baseline estimates. Column 1 includes neither controls nor fixed effects and yields an LTCI coefficient of 0.9676. Column 2 adds individual, household, and city-level controls, reducing the coefficient to 0.4220. Columns 3 and 4 introduce individual and year fixed effects; their policy coefficients are 0.1858 and 0.1860, respectively, and both are significant at the 1% level. Because the outcome is the ratio of tertiary- to secondary-industry value added, the preferred estimate means that LTCI raises this ratio by an average of 0.186 units. Relative to the sample mean of 1.6422, the absolute change is approximately 11.3%; it should not be interpreted as a direct 18.6% increase in industrial structure. The magnitude therefore reflects economically meaningful structural adjustment and supports H1.

**Table 2:** LTCI pilots and industrial structure upgrading.

Variable	(1)	(2)	(3)	(4)
LTCI pilot	0.9676***	0.4220**	0.1858***	0.1860***
	(0.1034)	(0.1869)	(0.0387)	(0.0403)
Individual and household controls	No	Yes	No	Yes
City-level controls	No	Yes	No	Yes
Individual fixed effects	No	No	Yes	Yes
Year fixed effects	No	No	Yes	Yes
Observations	2,498	2,326	1,334	1,231
Individuals	1,701	1,589	537	494
Cities	116	108	80	73
R^2^/within R^2^	0.4186	0.6888	0.1778	0.3290

City-clustered robust standard errors are shown in parentheses. *** and ** denote significance at the 1% and 5% levels, respectively. Columns 3 and 4 report within R^2^.

### 4.2 Robustness checks

#### 4.2.1 Parallel-trends test

**Figure 1:**
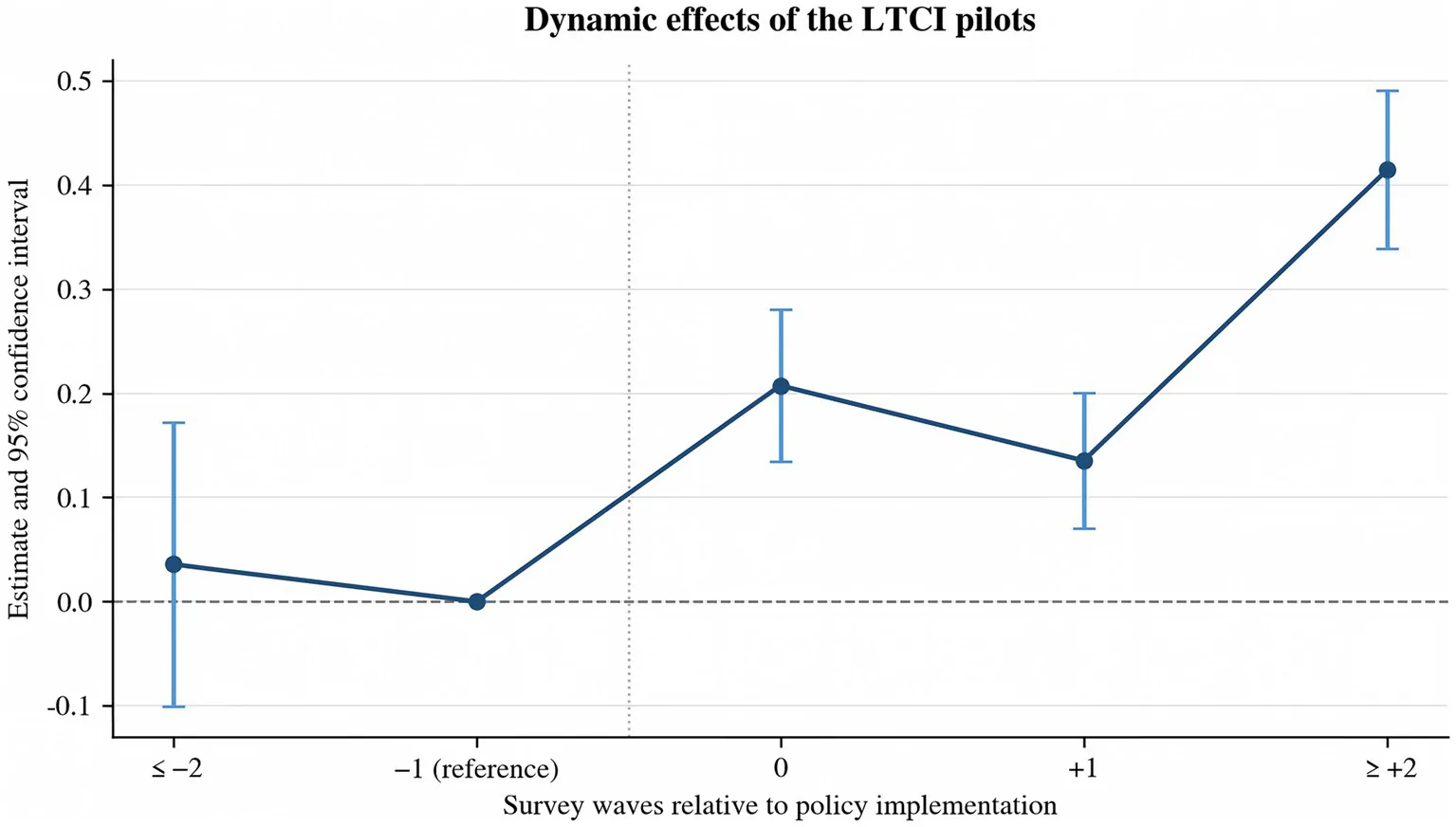
Parallel trends and dynamic effects of the LTCI pilots. The most recent pre-policy survey wave is the reference period. Error bars show 95% confidence intervals. The model includes the full control set and individual and year fixed effects, and standard errors are clustered by city.

The CFPS is conducted every two years, and city implementation dates do not always coincide with survey years. To ensure a consistent interpretation of relative time, the second and earlier pre-policy survey waves are pooled, and the most recent pre-policy wave is the reference period. Figure 1 shows that the pooled pre-policy coefficient is 0.0357 (standard error 0.0696; *p* = 0.609), indicating a common pre-policy trajectory for pilot and nonpilot cities. The coefficients in the implementation wave, the first post-policy wave, and the second and later post-policy waves are 0.2075, 0.1354, and 0.4147, respectively, and are all positive at the 1% level. This pattern shows that the policy effect persists and strengthens after implementation.

A complementary regression using only pre-policy observations estimates a linear differential trend of 0.0281 (standard error 0.0378; *p* = 0.461). The pooled-wave and linear pre-trend tests therefore provide consistent evidence in support of the parallel-trends assumption.

#### 4.2.2 Staggered-adoption bias check

When treatment timing differs across cohorts and treatment effects are heterogeneous, a conventional two-way fixed-effects model may use already-treated units as controls for later-treated cohorts, producing comparisons that are difficult to interpret (24). We therefore apply the Callaway–Sant’Anna multiperiod difference-in-differences estimator, use never-treated cities as the comparison group, estimate group-time average treatment effects, and aggregate them according to the treated sample. Standard errors are clustered by city. The overall average treatment effect on the treated is 0.1974 (standard error 0.0237; *p* < 0.001), which is close to the baseline coefficient of 0.1860. The effects in the policy wave, the first post-policy wave, and the second or later post-policy waves are 0.2052, 0.1113, and 0.3804, respectively, with standard errors of 0.0279, 0.0243, and 0.0258; all are significant at the 1% level. The identifiable pre-policy placebo effect is 0.1025 (standard error 0.0794; *p* = 0.204), indicating no systematic group-time difference before reform. The conclusion therefore remains stable after accounting for staggered adoption and heterogeneous treatment effects.

#### 4.2.3 Propensity-score-matched difference-in-differences

Pilot selection may be related to pre-existing economic development and public-service capacity. Propensity scores are therefore estimated using 2014 city-level GDP per capita, urbanization, GDP growth, financial development, and informatization. The analysis applies 10-nearest-neighbor matching with replacement on the logit index and re-estimates the difference-in-differences model in the matched panel using average-treatment-effect-on-the-treated weights. All 13 pilot cities and 35 distinct control cities remain after matching.

Table 3 shows that matching substantially improves comparability. The mean absolute standardized difference falls from 0.6649 to 0.0581, and the maximum absolute standardized difference declines to 0.0993. The PSM-DID estimate in Table 4 is 0.1426 and remains significant at the 1% level.

**Table 3:** Standardized differences before and after propensity-score matching.

Matching covariate	Before matching	After matching
Log city GDP per capita	1.2897	0.0294
Urbanization rate	0.9899	0.0993
City GDP growth rate	0.2821	0.0369
City financial development	0.3725	0.0850
City informatization	−0.3902	0.0400
Mean absolute standardized difference	0.6649	0.0581
Maximum absolute standardized difference	1.2897	0.0993

Standardized differences divide the treatment–control mean difference by the pooled standard deviation. After matching, all absolute standardized differences are below 0.10.

**Table 4:** Robustness checks.

Specification	DID coefficient	SE	*N*	Within R^2^
Baseline	0.1860***	0.0403	1,231	0.3290
PSM-DID (ATT weighted)	0.1426***	0.0464	1,049	—
Log industrial structure upgrading	0.0306***	0.0112	1,231	—
Excluding 2020	0.2035***	0.0374	857	0.6237

All specifications include the full control set, excluding the industrial structure control, as well as individual and year fixed effects. Standard errors are clustered by city. *** denotes significance at the 1% level.

#### 4.2.4 Alternative outcome and sample

When the dependent variable is replaced with ln(1 + industrial structure upgrading index), the policy coefficient is 0.0306 and remains significant at the 1% level. The transformation exp (0.0306) − 1 corresponds to an approximate relative change of 3.11% on this logarithmic scale. Because the COVID-19 pandemic may have affected service supply, population mobility, and urban economic structure simultaneously, the 2020 observations are also excluded. The resulting coefficient is 0.2035 and remains significant at the 1% level. These estimates retain the positive direction under different outcome definitions and samples.

#### 4.2.5 Random-assignment placebo test

**Figure 2:**
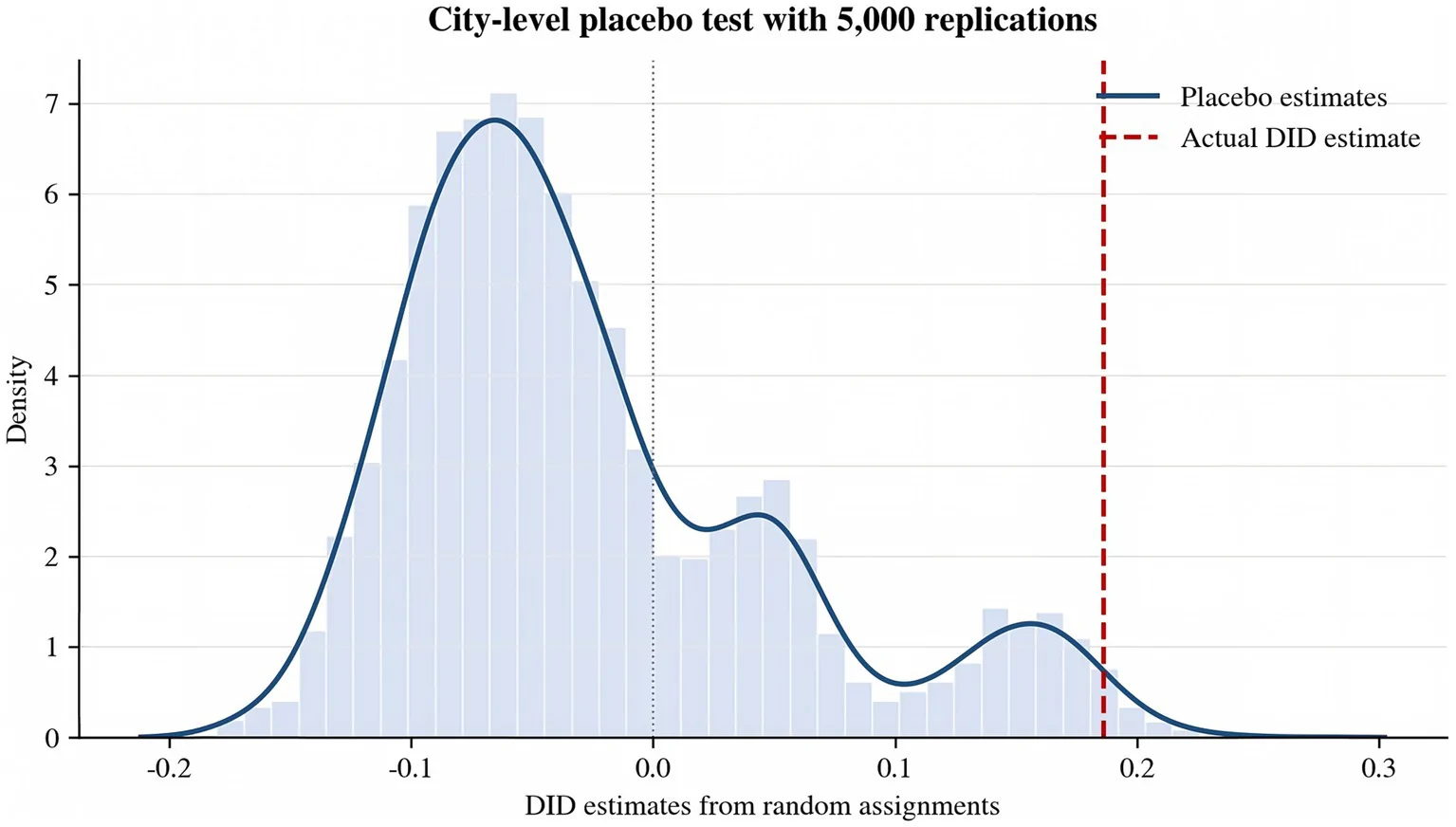
City-level random-assignment placebo test with 5,000 replications. The dashed red line marks the actual DID coefficient of 0.1860. Random assignments preserve the actual number of pilot cities in each policy cohort. Random seed: 20260807.

Fictitious pilot cities and policy cohorts are randomly assigned among the 73 cities in the baseline sample while preserving the actual number of pilot cities in each cohort. The model is re-estimated 5,000 times. The actual DID coefficient is 0.1860, whereas the placebo estimates have a mean of −0.0260 and a standard deviation of 0.0799. Only 62 of the 5,000 simulated absolute coefficients are at least as large as the actual estimate, producing a two-sided empirical *p*-value of 0.0124. Figure 2 places the actual estimate in the right tail of the random-assignment distribution. Together with the parallel-trends test, the Callaway–Sant’Anna estimates, PSM-DID, the alternative outcome, and the exclusion of 2020, this result shows that the finding is not driven by a particular comparison group, functional form, or sample period.

## 5 Heterogeneity analysis

Policy heterogeneity is examined using pre-policy city characteristics from 2014. Government intervention and regional location are evaluated through group interactions, while financial development is entered as a standardized continuous interaction. Marginal policy effects are calculated at one standard deviation below and above the mean of financial development. Table 5 reports the results.

**Table 5:** Heterogeneous effects of the LTCI pilots.

Dimension	Group/interaction	Policy effect	SE	*p*-value
Government intervention	Low intervention	−0.0250	0.0464	0.592
	High intervention	0.2436***	0.0339	<0.001
	Between-group difference	0.2686***	0.0508	<0.001
Region	Non-eastern	−0.0465	0.0627	0.461
	Eastern	0.1967***	0.0411	<0.001
	Between-group difference	0.2432***	0.0736	0.0016
Financial development	Low (−1 SD)	−0.1246	0.1053	0.241
	High (+1 SD)	0.1205***	0.0414	0.0050
	LTCI × financial development	0.1225***	0.0399	0.0031

Government intervention is split at the 2014 city median; eastern China is classified by province; and financial development is standardized using its 2014 value. All models include the full control set and individual and year fixed effects, with standard errors clustered by city. *** denotes significance at the 1% level. The effective sample contains 1,197 observations, 478 individuals, and 64 cities.

### 5.1 Government intervention

In cities with high government intervention, the LTCI coefficient is 0.2436 and is significant at the 1% level. The difference in policy effects between high- and low-intervention cities is 0.2686 (*p* < 0.001). Local implementation capacity in financing coordination, provider entry, disability assessment, service supervision, and interdepartmental cooperation therefore improves the conversion of insurance payments into service expansion and industrial upgrading, consistent with H4.

### 5.2 Eastern and non-eastern regions

The policy coefficient for eastern cities is 0.1967 and is significant at the 1% level. The difference between eastern and non-eastern cities is 0.2432 (*p* = 0.0016). Better medical and eldercare resources, greater population and service-demand density, and more mature service markets allow insurance payments to generate faster provider entry and health-service expansion in eastern China, consistent with H4.

### 5.3 Financial development

The interaction between LTCI and financial development is 0.1225 and is significant at the 1% level. At one standard deviation above the mean of financial development, the policy effect is 0.1205 and is also significant at the 1% level. Financial development eases the financing constraints of care institutions and health-service firms and increases the ability of private capital to respond to insurance-supported demand, consistent with H4.

## 6 Mechanism analysis

A two-step mechanism approach is used. After the baseline model establishes that LTCI promotes industrial structure upgrading, current employment and the logarithm of the total value of household financial products are used as mechanism outcomes. The same common sample, covariates, individual and year fixed effects, and city-clustered standard errors are applied. When a mechanism variable is the dependent variable, it is removed from the corresponding control set. Table 6 reports the results.

**Table 6:** Mechanism analysis.

Variable	Currently employed	ln(1 + financial product value)
LTCI pilot	0.0704**	0.4125***
	(0.0322)	(0.1555)
Individual and household controls	Yes	Yes
City-level controls	Yes	Yes
Individual fixed effects	Yes	Yes
Year fixed effects	Yes	Yes
Observations	1,231	1,231
Individuals	494	494
Cities	73	73

City-clustered robust standard errors are shown in parentheses. A mechanism variable is excluded from the corresponding control set when it is used as the dependent variable. *** and ** denote significance at the 1 and 5% levels, respectively.

### 6.1 Long-term care insurance releases household labor supply

With current employment as the mechanism outcome, the LTCI coefficient is 0.0704 (standard error 0.0322) and is significant at the 5% level, implying an average increase of approximately 7.04 percentage points in household members’ employment probability. By converting part of informal family care into institutional, community-based, and home-based formal services, LTCI releases time previously absorbed by caregiving and facilitates re-entry into paid employment. The result is consistent with prior evidence on labor supply (12–14) and supports the transmission chain from formal-care substitution to released labor supply and reallocation toward market employment. H2 is supported.

### 6.2 Long-term care insurance improves household financial asset allocation

With ln(1 + total value of financial products) as the mechanism outcome, the LTCI coefficient is 0.4125 (standard error 0.1555) and is significant at the 1% level. The transformation exp.(0.4125) − 1 corresponds to an approximate 51.1% change on the ln(1 + x) scale. By sharing disability-related care expenditure and stabilizing expectations of future costs, LTCI allows households to reallocate funds from purely liquid reserves toward a broader set of financial products. The resulting demand for financial services and supply of longer-term funds can support the expansion of health, eldercare, and related modern service industries. This evidence supports the transmission chain from risk sharing to stable expectations, improved financial asset allocation, and industrial structure upgrading, and therefore supports H3.

## 7 Conclusion and policy implications

Using CFPS individual panel data for 2014–2020, city-level macroeconomic indicators, and LTCI pilot information, this study reaches three main conclusions. First, LTCI raises the ratio of tertiary- to secondary-industry value added by an average of 0.186 units, approximately 11.3% of the sample mean. The Callaway–Sant’Anna overall average treatment effect on the treated is 0.1974, confirming that the conclusion remains stable after accounting for staggered adoption and heterogeneous treatment effects. Second, the structural effect is stronger where government coordination is greater, in eastern cities with stronger service-supply foundations, and where financial development is higher. Third, LTCI raises household members’ employment probability by approximately 7.04 percentage points and substantially improves household financial product allocation, identifying released labor supply and improved household capital allocation as two important transmission channels.

The findings have several policy implications. First, LTCI should be developed from the dual perspective of social protection and industrial development. Stable financing and payment standards can strengthen market expectations and support sustainable nursing, rehabilitation, and community-care supply. Second, policy design should make full use of LTCI’s labor-supply effect by expanding formal home- and community-based care, improving referral services, and linking family caregivers to employment services and vocational training. Third, interdepartmental coordination should be strengthened and regional support differentiated: eastern cities can deepen specialized care, rehabilitation, and smart-health services, while central and western cities can build service capacity through fiscal support, workforce development, and cross-regional cooperation. Fourth, financial institutions can develop prudent household financial products suited to long-term care needs and credit products based on LTCI settlement cash flows, thereby improving household intertemporal risk management and mobilizing long-term capital for eldercare and health services.

This study has several limitations. LTCI pilots were not assigned completely at random, and time-varying city factors may remain even after the parallel-trends test, the Callaway–Sant’Anna estimator, PSM-DID, and the random-assignment placebo test. The city-level industrial structure measure cannot separately identify the contributions of nursing care, rehabilitation, health management, and other specific industries. In addition, the number of CFPS waves that can be matched to complete city controls is limited, constraining the identification of longer-run dynamic effects. Future research can combine longer city panels with provider registration, industry employment, and capital-investment data and can distinguish the effects of different financing levels, benefit packages, and payment arrangements.

## Data Availability

The individual- and household-level data analyzed in this study are available from the China Family Panel Studies subject to registration and its terms of use. City-level indicators were compiled from publicly available statistical yearbooks and statistical communiques. The processed analytical dataset and replication code are available from the corresponding author upon reasonable request, subject to the data provider’s conditions.

## References

[1] Lilly MB Laporte A Coyte PC. Labor market work and home care's unpaid caregivers: a systematic review of labor force participation rates, predictors of labor market withdrawal, and hours of work. Milbank Q. (2007) 85:641–90. doi: 10.1111/j.1468-0009.2007.00504.x, 18070333 PMC2690351

[2] Van Houtven CH Norton EC. Informal care and health care use of older adults. J Health Econ. (2004) 23:1159–80. doi: 10.1016/j.jhealeco.2004.04.008, 15556241

[3] Carmichael F Charles S. The opportunity costs of informal care: does gender matter? J Health Econ. (2003) 22:781–803. doi: 10.1016/S0167-6296(03)00044-4, 12946459

[4] Heitmueller A. The chicken or the egg? Endogeneity in labour market participation of informal carers in England. J Health Econ. (2007) 26:536–59. doi: 10.1016/j.jhealeco.2006.10.005, 17098311

[5] Yue W Wang X Zhang Q. Health risks, medical insurance, and household financial vulnerability. China Ind Econ. (2021) 10:175–92. doi: 10.19581/j.cnki.ciejournal.2021.10.009

[6] General Office of the Ministry of Human Resources and Social Security of the People's Republic of China. Guiding Opinions on Launching Pilot Programs for the Long-Term Care Insurance System (Ren She Ting Fa [2016] No. 80). Beijing: General Office of the Ministry of Human Resources and Social Security of the People’s Republic of China (2016).

[7] National Healthcare Security Administration, Ministry of Finance of the People's Republic of China. Guiding Opinions on Expanding Pilot Programs for the Long-Term Care Insurance System (Yi Bao Fa [2020] No. 37). Beijing: National Healthcare Security Administration of the People’s Republic of China and Ministry of Finance of the People’s Republic of China (2020).

[8] Feng J Wang Z Yu Y. Does long-term care insurance reduce hospital utilization and medical expenditures? Evidence from China. Soc Sci Med. (2020) 258:113081. doi: 10.1016/j.socscimed.2020.113081, 32540515

[9] Chen H Ning J. The impacts of long-term care insurance on health care utilization and expenditure: evidence from China. Health Policy Plan. (2022) 37:717–27. doi: 10.1093/heapol/czac003, 35032390

[10] Lei X Bai C Hong J Liu H. Long-term care insurance and the well-being of older adults and their families: evidence from China. Soc Sci Med. (2022) 296:114745. doi: 10.1016/j.socscimed.2022.114745, 35093795

[11] Liu H Ma J Zhao L. Public long-term care insurance and consumption of elderly households: evidence from China. J Health Econ. (2023) 90:102759. doi: 10.1016/j.jhealeco.2023.102759, 37146408

[12] Fu R Noguchi H Kawamura A Takahashi H Tamiya N. Spillover effect of Japanese long-term care insurance as an employment promotion policy for family caregivers. J Health Econ. (2017) 56:103–12. doi: 10.1016/j.jhealeco.2017.09.011, 29040896

[13] Yu X Huang J Kang Z Yu W. Old-age care security and female labor force participation: a policy evaluation based on China's rural long-term care insurance pilots. Chin Rural Econ. (2021) 11:125–44.

[14] Yang S Xing H. The labor supply effect of long-term care insurance in China: an analysis of different care models. Econ Manag Res. (2025) 7:184–91. doi: 10.19851/j.cnki.CN11-1010/F.2025.07.249

[15] Huang Z Jian A Wang J Chen H. The effects of public long-term care insurance on the long-term care industry in China: a quasi-experimental study. BMC Geriatr. (2025) 25:276. doi: 10.1186/s12877-025-05933-6, 40281486 PMC12023454

[16] Guo M Li Y Zhang W. Supply expansion of long-term services and supports: evidence from China's long-term care insurance pilot. J Econ Behav Organ. (2025) 239:107267. doi: 10.1016/j.jebo.2025.107267

[17] Ngai LR Pissarides CA. Structural change in a multisector model of growth. Am Econ Rev. (2007) 97:429–43. doi: 10.1257/aer.97.1.429

[18] Buera FJ Kaboski JP. The rise of the service economy. Am Econ Rev. (2012) 102:2540–69. doi: 10.1257/aer.102.6.2540

[19] Besley T Persson T. The origins of state capacity: property rights, taxation, and politics. Am Econ Rev. (2009) 99:1218–44. doi: 10.1257/aer.99.4.1218

[20] Faguet JP. Does decentralization increase government responsiveness to local needs? Evidence from Bolivia. J Public Econ. (2004) 88:867–93. doi: 10.1016/S0047-2727(02)00185-8

[21] Rajan RG Zingales L. Financial dependence and growth. Am Econ Rev. (1998) 88:559–86.

[22] Wurgler J. Financial markets and the allocation of capital. J Financ Econ. (2000) 58:187–214. doi: 10.1016/S0304-405X(00)00070-2

[23] Hsu PH Tian X Xu Y. Financial development and innovation: cross-country evidence. J Financ Econ. (2014) 112:116–35. doi: 10.1016/j.jfineco.2013.12.002

[24] Callaway B Sant'Anna PHC. Difference-in-differences with multiple time periods. J Econom. (2021) 225:200–30. doi: 10.1016/j.jeconom.2020.12.001

[25] Sun L Abraham S. Estimating dynamic treatment effects in event studies with heterogeneous treatment effects. J Econom. (2021) 225:175–99. doi: 10.1016/j.jeconom.2020.09.006

[26] Abadie A Athey S Imbens GW Wooldridge JM. When should you adjust standard errors for clustering? Q J Econ. (2023) 138:1–35. doi: 10.1093/qje/qjac038

[27] Lei X. China's long-term care security system for older adults under a social welfare policy analysis framework. China Labour. (2025) 5:72–88. doi: 10.19390/j.cnki.chinalabor.2025.05.006

[28] Campbell JC Ikegami N Gibson MJ. Lessons from public long-term care insurance in Germany and Japan. Health Aff. (2010) 29:87–95. doi: 10.1377/hlthaff.2009.0548, 20048365

[29] Yang F He Z. Intersectoral effects and development drivers of China's health industry: an input–output analysis. World Surv Res. (2026) 1:66–81. doi: 10.13778/j.cnki.11-3705/c.2026.01.005

[30] Palumbo MG. Uncertain medical expenses and precautionary saving near the end of the life cycle. Rev Econ Stud. (1999) 66:395–421. doi: 10.1111/1467-937X.00092

[31] De Nardi M French E Jones JB. Why do the elderly save? The role of medical expenses. J Polit Econ. (2010) 118:39–75. doi: 10.1086/651674

[32] Sugawara S Nakamura J. Can formal elderly care stimulate female labor supply? The Japanese experience. J Jpn Int Econ. (2014) 34:98–115. doi: 10.1016/j.jjie.2014.05.006

[33] Løken KV Lundberg S Riise J. Lifting the burden: formal care of the elderly and labor supply of adult children. J Hum Resour. (2017) 52:247–71. doi: 10.3368/jhr.52.1.0614-6447R1

[34] An R Tang X Qi S Wang Z Cui L Zhang H . Care needs and their determinants among community-dwelling older adults: a baseline survey in three provinces. Chin Gen Pract. (2025) 28:59–64. doi: 10.12114/j.issn.1007-9572.2023.0628

[35] Zhu J Cheng Y Li W. Equalization of basic public services, industrial structure upgrading, and the urban–rural income gap. J Hunan Univ Sci Technol Soc Sci Ed. (2025) 28:146–55. doi: 10.13582/j.cnki.1672-7835.2025.04.017

[36] Duarte M Restuccia D. The role of structural transformation in aggregate productivity. Q J Econ. (2010) 125:129–73. doi: 10.1162/qjec.2010.125.1.129

